# Swelling and Antimicrobial Activity Characterization of a GO-Reinforced Gelatin—Whey Hydrogel

**DOI:** 10.3390/gels9010018

**Published:** 2022-12-27

**Authors:** Pompilia Mioara Purcea Lopes, Dumitriţa Moldovan, Radu Fechete, Doina Prodan, Carmen Rodica Pop, Ancuța M. Rotar, Violeta Popescu

**Affiliations:** 1 Physics and Chemistry Department, Technical University of Cluj-Napoca, 28 Memorandumului Str., 400114 Cluj-Napoca, Romania; 2 Composite Materials Department, Chemistry Research Institute “Raluca Ripan”, “Babes-Bolyai” University, 30 Fantanele Str., 400294 Cluj-Napoca, Romania; 3 Department of Food Science, Faculty of Food Science and Technology, University of Agricultural Sciences and Veterinary Medicine Cluj-Napoca, 64 Calea Floresti Str., 400509 Cluj-Napoca, Romania

**Keywords:** whey, graphene oxide, gelatin, crosslinking, swelling, NMR relaxometry, antibacterial activity

## Abstract

Whey-based hydrogel samples with increasing concentrations of graphene oxide (GO) were studied, against a control sample (M), for swelling behavior in light of nuclear magnetic resonance (NMR) and mathematical models of the diffusion process and for antibacterial activity. Graphene oxide (GO) is an optimal filler for whey-based hydrogels, giving them improved mechanical and swelling properties at low concentrations. Crosslinking induces a certain stiffness of the hydrogels, which is why only the first part of the swelling process (<60%) follows the first-order model, while during the whole time interval, the swelling process follows the second-order diffusion model. The NMR relaxometry results are consistent with the swelling behavior of GO-reinforced whey–gelatin composite hydrogels, showing that higher GO concentrations induce a higher degree of cross-linking and, therefore, lower swelling capacity. Only hydrogel samples with higher GO concentrations demonstrated antibacterial activity.

## 1. Introduction

Graphene oxide is studied in many kinds of research to improve the functional properties of hydrogels [1,2,3]. For food packaging applications, graphene oxide is usually obtained with the modified Hummers’ method [4] which is more suitable because of the lower content of toxic gases released during the procedure. The numerous functional groups of oxygen, epoxy, hydroxyl, carbonyl, and carboxyl on the layered surface of graphene oxide [4,5] easily create bonds with a wide category of substances, demonstrating their efficiency in transport systems, wastewater treatment [6], free lactose dairy product technologies [5], antimicrobial properties [7], food packaging [8,9], and antimicrobial packaging [10,11]. An important number of research studies point out that hydrogels based on biopolymers reinforced with graphene derivates (graphene oxide, reduced graphene oxide, chemically modified graphene) are a sustainable resource for food packaging applications, reducing waste and increasing food safety [4].

Whey is an abundant and low-cost byproduct of the dairy industry, rich in globular proteins that can form gels. The merit of whey in nutritional supplements, functional foods, transport, and controlled release systems has been well-known for decades. Moreover, its versatility in blending with other polymers and nanoparticles allows the improvement of its plastic properties [12,13] as an ecofriendly and biocompatible alternative to petroleum materials used in the food packaging industry, such as antimicrobial packaging [7,14,15,16,17], as a moisture and oxygen barrier [18], and as oxygen scavengers [19]. Therefore, innovative whey- or gelatin-based nanocomposite hydrogels were developed by crosslinking with TiO_2_ nanoparticles [18], cellulose nanofibers [20,21], copper compounds [22,23,24], and montmorillonite [25] to meet consumer demands for healthier and fresher food.

Fibrillar proteins contained in collagen, another food industry by-product, are converted to gelatin by heat denaturation. Its high gel-forming ability has captured the attention of researchers for many decades in the optimization of composite hydrogels with applications in medicine [26] as an ecofriendly alternative precursor, along with the other major group, carbohydrates [27,28,29]. The addition of gelatin to other protein-based gels is a common method of modifying texture, stiffness and water holding capacity e.g., egg white protein gels [30] and whey protein gels [31].

Food packaging is no longer just a simple layer that protects food from light and impurities during storage and transport. On the one hand, consumers are more and more aware that the food they eat needs to meet high standards of quality, freshness, nutritional value, and low processing, and, on the other hand, advanced technological lines allow the commercialization of a very large quantity of food, which has to keep the aforementioned qualities for a longer period of time. In the light of these two aspects this study proposes a new type of nanocomposite hydrogel based on whey and gelatin reinforced with graphene oxide, as the latter has demonstrated both good swelling behavior in most hydrogel formulations due to its high surface area and homogenous dispersion of the nanoparticles in the polymer matrix, and antibacterial activity. The new hydrogel formula merges the attributes of whey with those of gelatin and graphene oxide, demonstrating high potential as absorbent pads in high water content food packaging systems. The properties of whey–gelatin and graphene oxide hydrogels were investigated by nuclear magnetic resonance (NMR) for their swelling behavior and antibacterial activity.

As recently observed by other authors, there are few studies characterizing the properties of composite hydrogels prepared from whey–gelatin combinations, although these precursors have demonstrated utility in a wide range of fields [32]. Therefore, further exploration of this hydrogel system may offer promising benefits.

## 2. Results and Discussion

### 2.1. Swelling Behavior

**Figure 1:**
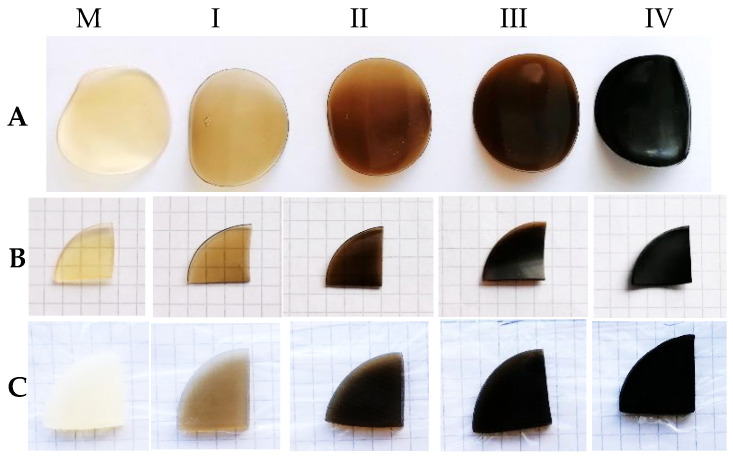
Hydrogel samples after drying in figure (**A**), being prepared for swelling in figure (**B**), and after 48 h of swelling in figure (**C**); I, II, III, and IV with increasing GO concentration, and the control sample M.

The dry composite hydrogel samples are shown in Figure 1A,B. As can be seen, there is a tendency to contract, which rises with increasing graphene oxide concentration. As expected, all samples preserved their integrity better than the control sample throughout the swelling process. In this regard, GO confers improved plastic properties to whey–gelatin-based hydrogels, giving them resistance in wet environments. These qualities are confirmed by other authors in their studies for food packaging based on graphene derivates [4,33].

One of the essential properties of hydrogels is their swelling capacity in wet environments. Indirectly, the swelling degree can be an indicator of the crosslinking achieved in the hydrogel formation process [9].

**Figure 2:**
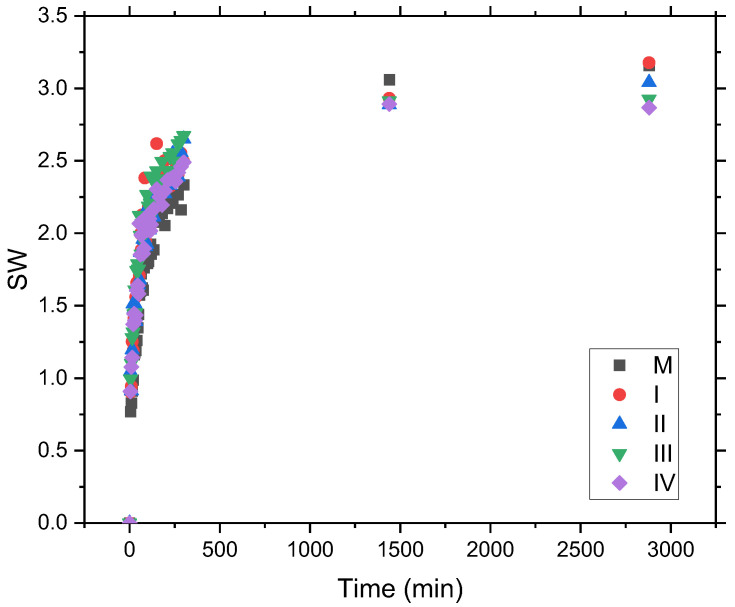
The curve of swelling degree as a function of time for hydrogel samples with increasing GO concentration (I, II, III, and IV) and control sample M.

To determine the swelling degree of the hydrogel samples, Equation (1) was used. The largest amount of water was absorbed in the first 5 h and, as can be seen in Figure 2, the samples with GO demonstrated better swelling capacity compared with the control sample. However, in the next part of the swelling process, the swelling degree is inversely proportional to the concentration of GO. Other studies [34] have found that the effect of crosslinking is the reason why the oxidized graphene enhanced the rigidity of hydrogels corresponding to its concentration in the hydrogel formula [35]. The strengthening of the hydrogel samples induced by GO incorporation determines their integrity in the hydration medium even after 2 days (see Figure 1) with a consequent decrease in the swelling capacity, as can be seen in Table 1, due to the barrier created by the strongly anisotropic 2D structure of the GO layers in the more open 3D porous protein matrix. Similar results were found in other studies [35]. In conclusion, sample I, with minimal GO content, showed the best hydration capacity at the end of the time interval analyzed (see Table 1).

**Figure 3:**
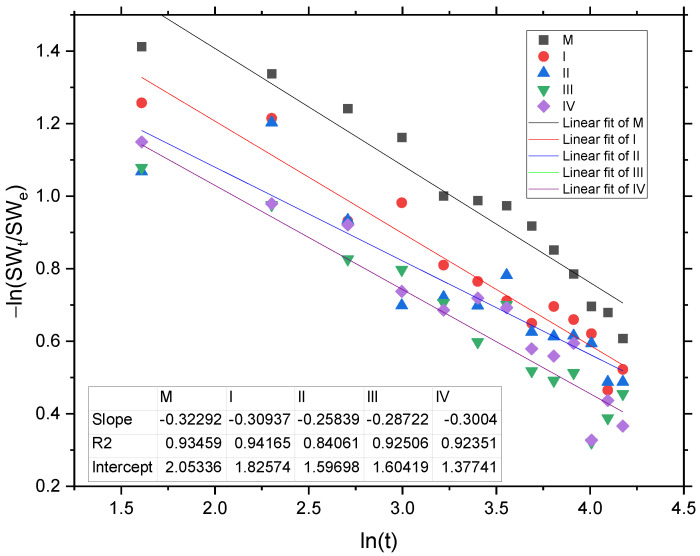
The plot of −*ln (SWt/SWe)* against *ln (t)* for control sample (M) and the ones containing increasing concentrations of graphene oxide (I, II, III, and IV) for swelling degree < 60%.

Further, the type of diffusion characterizing the swelling process was investigated. The Fickian diffusion model was evaluated by the power law Equation (2) plotted in Figure 3. From the linearized equations, one can conclude that the diffusion coefficient *n* has values in the range 0 < *n* ≤ 0.5; thus, the first 60% of diffusion process follows the first order Fickian model, being a time-dependent penetration of the waterfront through the hydrogel matrix. Authors observed similar swelling behavior of hydrogels based on whey and gelatin crosslinked with copper sulphate in their previous works [21,33], except for the diffusion constant K, which, unlike in previous studies, is decreasing with increasing GO, though the sample with minimum GO content (I) has the K value closest to the control sample (M) due to the lower crosslinking degree.

**Figure 4:**
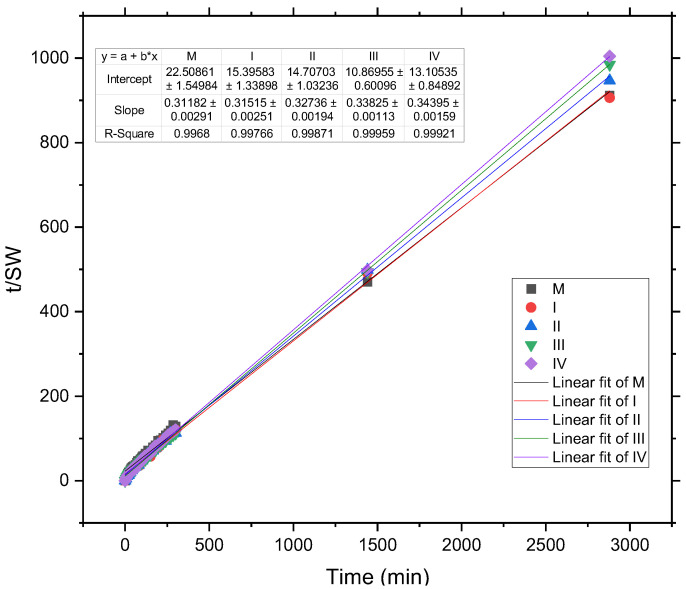
Graphical representation of the Schott equation for hydrogel samples with (I, II, III, and IV) and without GO (M).

The diffusion process over the whole time interval follows the non-Fickian model, of order two, evaluated with Schott’s equation, as represented in Figure 4.

From Figure 4, one can clearly see the linear dependence of t/SW and t in Schott’s equation, which indicates that the swelling process follows a second-order, non-Fickian diffusion for the whole-time interval. From the slope of Schott’s equation, the theoretical values of swelling degree and the diffusion constant K were obtained, as in Equations (7) and (8), and are presented in Table 2. The calculated and experimental SW_e_ values are almost equal for the hydrogels with GO and are slightly different for the control sample (M). These results confirm the cross-linking action of GO in the hydrogel samples resulting in a better stability in the swelling medium, but a lower swelling degree compared to the control sample. In light of this findings, the lower GO concentration hydrogel exhibits adequate features for a moisture absorbent pad in food packaging systems.

### 2.2. H NMR Relaxometry

**Figure 5:**
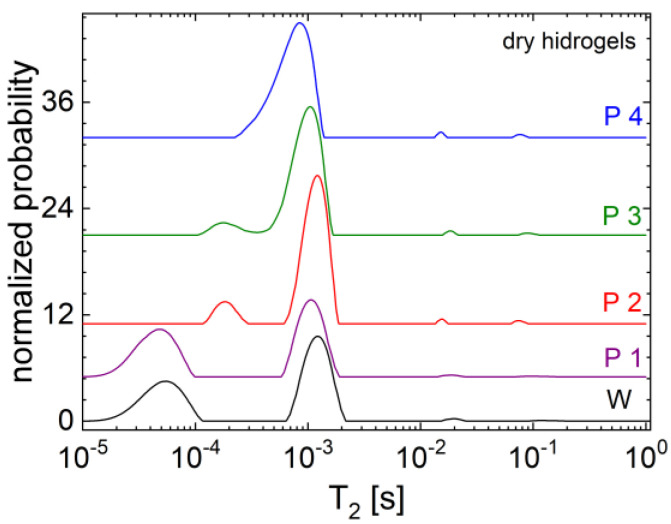
The T2 distributions of dry hydrogel samples containing 0 (black), 1× (purple), 2× (red), 3× (olive), 4× (blue) graphene.

The ^1^H NMR relaxometry is a powerful method that can be used for the characterization of the dynamics of various materials containing protons, such as hydrogels [23,36]. The distribution of transverse relaxation times T_2_ measured for the dry hydrogels, control sample (M), and those with graphene oxide (GO) with increasing concentration is presented in Figure 5. One can observe four peaks distributed from lower relaxation time T_2_ values, such as tens of microseconds for the control sample and the hydrogel with the smallest amount of GO, to higher T_2_ values, such as approximately 100 ms. These peaks located at higher T_2_ values can be attributed to dangling free ends of biopolymers forming the hydrogel. The main peak (characterized by the largest area under the curve) is located for all samples around 1 ms (1.22 ms for the control sample and 0.84 ms for sample IV). These T_2_ values are specific to elastomers, such as natural rubber [36,37], which is more elastic compared to our hydrogels. The rigidity of the produced hydrogel is probably caused by the components characterized with even lower T_2_ values, which are characteristic to plastic-type materials. In this sense, one can observe, by comparing the T_2_ distributions presented in Figure 5, that a small amount of graphene oxide, as in the case of sample I, will not influence the microscopic dynamics too much, as the T_2_ distributions measured for the dry control sample and I are similar. One can observe a slight shift of peaks located at lower T_2_ values from ~54.6 μs for control sample M to ~47.9 μs for the sample I and from 1.22 ms for sample M to 1.06 ms for sample I. At a visual inspection, the stiffness of samples increased with the increase in the graphene oxide (GO) content, therefore, the most rigid sample is labeled as IV. Furthermore, since the samples II, III, and IV are not characterized by peaks located below 100 μs, one can conclude that such peaks observed for the samples M and I can be associated with extremely rigid components but which are spatially less extended. Most probably the peaks located between 100 μs and 2 ms can be associated to ^1^H located in long-range cross-linked polymer chains.

The most noticeable effect of graphene oxide (GO) is as follows: (i) to participate in the formation of additional cross-links resulting in an increased stiffness of the hydrogels reflected from the shift of the main peak to lower T_2_ values and (ii) to connect the hard cores (deduced from the presence of a significant peak located at T_2_ values lower than 100 μs for samples M and I) to the long chains, as can be observed from the migration of the peak located at lower T_2_ values from 47.9 μs for the sample I, to 179 μs for the samples II and III, and to ~450 μs for the sample IV. In the last case, it seems that the largest amount of graphene oxide (GO) was able to incorporate the hard core described before to the long cross-linked polymer chains. This conclusion was supported by the observation that the peak characterizing the most rigid components migrated toward a larger value so much that it is observed as a left shoulder of the main peak. In each distribution one can observe another peak located at ~20 ms, characterizing some semi-mobile components. It can be seen that this peak is not significantly affected by the presence or the amount of graphene oxide, with the exception of the peaks’ width, which is narrower in the case of hydrogels with an increased amount of GO, e.g., samples II, III, and IV.

**Figure 6:**
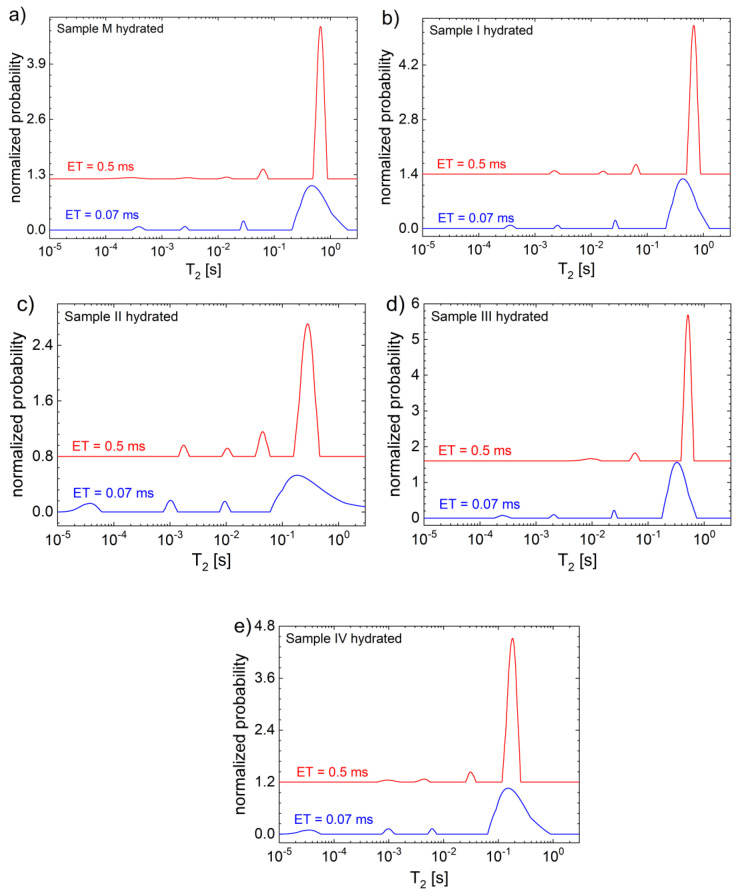
The T_2_ distributions of hydrated hydrogel samples; (**a**) control sample M, (**b**) sample I, (**c**) sample II, (**d**) sample III, and (**e**) sample IV measured for an echo time ET = 0.07 ms (blue) and ET = 0.5 ms (red).

The T_2_ distributions measured for the hydrated samples are completely different compared to those of dry hydrogels (see Figure 6). The main peak is no longer located at T_2_ values of the order of 1 ms but are located at ~665 ms for the control sample and from ~678 ms for sample I to ~179 ms for sample IV, characterized by the largest amount of graphene oxide. For such samples containing a large amount of hydration water, it is customary to record the data (CPMG decays) with two echo times. The experimental data measured with an echo time TE = 0.07 ms (the smallest available) can clarify the T_2_ distributions at lover T_2_ values but cannot be used for clearly located peaks at larger T_2_ values (see the lower blue distribution in Figure 6). Conversely, experimental data measured with an echo time TE = 0.5 ms (see the upper red distribution in Figure 6) can clarify the T_2_ distributions at larger T_2_ values, but usually acts as a filter, and the peaks characterized by small T_2_ values are distorted or disappear. In all cases, the hydration water gives the main peak, and the position of this also reflects the stiffness of hydrogels being located at higher T_2_ values for the samples with no graphene oxide or with the lower amount of GO (see Figure 6a,b). With the increase in GO, the main peak is shifted towards lower T_2_ values, indicating that the main amount of ^1^H is located in less mobile components. Nevertheless, this trend is inverted for the samples II and III, probably due to sample II, where peaks also have significant amplitudes (and, hence, integral areas) at lower T_2_ values, while the rest of the samples present only peaks with smaller amplitudes located to the left of the main peak. As in the case of the dry sample, a small amount of GO will not significantly change the dynamics of polymer chains forming the hydrogel. The hydrated samples can be characterized by five peaks in the *T*_2_ distributions, as follows: (i) four of them are located at lower values and with a small amplitude (from ~30 μs for samples II and IV to 30.9–63 ms) reflecting the interaction between the cross-linked polymer network with water and a relatively low number of protons, respectively, and (ii) one located at larger T_2_ values reflecting the water adsorbed between the polymer chains into a restricted geometry. The main peak shift towards lower T_2_ values can be a reflection of the fact that the degree of cross-linking increased with the increase in graphene oxide particles, resulting in smaller pores (voids between polymer chains), but also can be a result of sample degradation. During hydration, in time the consistency of hydrogels is reduced and parts of these becomes free, being dissolved in water, leading to smaller T_2_ values due to an increase in the mobility of water molecules. The parts which are not dissolved can be seen as peaks appearing at much lower T_2_ values, as was expected for sample IV (see Figure 6e) but also observed for sample II (see Figure 6c).

### 2.3. Antimicrobial Activity

Following incubation on culture media inoculated with the bacterial strains under study, the hydrogel samples developed zones of inhibition whose dimensions can be seen in Table 3. Only the samples with the highest GO content showed bacterial inhibition activity against the tested strains. As in a previous author’s research [23], the control sample did not show any antibacterial activity against the tested strains. In this study the sample with the lowest GO content showed identical behavior to the control sample. This result is in congruence with the research of Barbolina et al., who established that at concentrations lower than 1 mg/mL, GO does not show any antibacterial activity against *E. coli* [11]. Another explanation could be that at the incubation temperature applied in this study (30 °C) the two strains have a slower multiplication rate. Hydrogel samples showing antibacterial activity were II, III, and IV. In general, the higher the concentration of GO, the stronger the inhibition of bacteria of the same strain type. The specificity of the inhibitory action towards certain strains is also observed, i.e., only samples III and IV show activity towards *Listeria monocytogenes* despite having high concentrations of GO. The best antibacterial activity was observed against Gram-negative strains, such as *Escherichia coli* and *Salmonella enteriditis*, in increasing direction of GO concentration.

In conclusion, GO induces selective antibacterial activity in hydrogel samples and at higher concentrations against *Escherichia coli* (II, III, and IV)*, Salmonella enteriditis* (II, III, and IV) and *Listeria monocytogenes* (III and IV). Current research highlights the antimicrobial activity of composite hydrogels containing graphene in combination with bivalent metals [38], essential oils [10], and polymers known for this type of behavior, such as chitosan [38]. These findings encourage further study to attribute antibacterial properties to the sample with the best swelling performance (I).

## 3. Conclusions

The GO is an optimal filler for gelatin–whey-based hydrogels, giving them improved stability throughout the hydration process and swelling properties at low concentrations. The swelling behavior of hydrogels was studied in light of ^1^H NMR relaxometry and mathematical models of the diffusion process, revealing results that correlate with each other and with the results of other studies. The GO causes a stiffening of the hydrogel in the direction of increasing concentration in the hydrogel matrix as an effect of cross-linking, an observation supported by the decrease in the degree of swelling of the composite hydrogels with increasing GO concentration, and by the higher T_2_ values of the control sample and the one with minimal GO content (sample I) with respect to the samples with higher GO concentration, as a consequence of the presence of ^1^H in rigid regions of the hydrogel matrix.

For optimal antibacterial activity, it is recommended to use a minimum concentration of 6.67 × 10^−3^% GO in the composition of hydrogels, in the absence of other antimicrobial agents. The cumulative effect of the three precursors, namely whey, gelatin, and GO, encourages the research to further improve this hydrogel formulation by impregnation with antimicrobial active substances.

This study brings a possible hydrogel formulation for moisture absorbent pads for fresh food with high moisture content packaging systems, with respect for current environmental concerns, such as sustainability, innovation, and recycling of waste from the food industry.

## 4. Materials and Methods

### 4.1. Materials

For the preparation of the hydrogel samples, GO was obtained by Hummer’s method by the Polymeric Composite Laboratory, Institute of Chemistry Raluca Ripan, Babes-Bolyai University, Cluj-Napoca, Romania; instant whey protein isolate (WPI) active was produced and distributed by Sly Nutritia SRL (Buzău, Romania); gelatin reagent (AMRESCO, LLC, Fountain Parkway Solon, Cleveland, OH, USA) and glycerol (Sigma-Aldrich, Taufkirchen, Germany) were used.

### 4.2. Hydrogel Preparation

Four suspensions of 0.001/0.005/0.01/0.05% GO (*w*/*w*) in distilled water were prepared, then kept in an ultrasound bath for 8 h for a good dispersion of GO. A total of 18 g of each GO suspension with different concentrations was mixed with 3 g of 5% (*w*/*w*) whey solution, 3 g of 1:1 (*w*/*w*) glycerin, and 3 g of gelatin. The control sample was brought to the same weight with distilled water (18 g). After that, the samples were kept in an ultrasound bath for 2 h at 45 °C for better homogenization. For clarity, the samples were marked as follows: M was the control sample containing no GO, and I, II, III, and IV were those with GO in the increasing order of the concentration. The final concentration of each precursor was 11.11% for gelatin, 0.56% for whey, 5.55% for glycerin, and the GO concentration corresponding to samples I, II, III, and IV were $0.66\times 10^{-3}$, $3.33\times 10^{-3}$, $6.67\times 10^{-3}$, and $33.33\times 10^{-3}$, respectively.

### 4.3. Swelling Kinetics

The swelling process took place by keeping the tea bags with samples in distilled water for specific time intervals (5 min in the first 2 h, then 15 min the next 3 h, and finally after 24 and 48 h), followed by weighing them after removing the excess of water with a paper towel. The weight of the dry hydrogels (W_2_), the wet tea bag (W_1_), and the swelled hydrogels (W_3_) can be found in the swelling degree (SW) Equation (1) [23,39,40].

(1) $$\mathrm{SW}=\frac{W_{3}-W_{2}-W_{1}}{W_{2}}$$

Equation (2) [23,39,41] was used to evaluate if the swelling process follows the Fickian diffusion, as follows:

(2) $$\frac{{\mathrm{SW}}_{t}}{{\mathrm{SW}}_{e}}={\mathrm{Kt}}^{n}$$

where SW_t_ and SW_e_ represent the swelling degree of the hydrogel at time *t* and at equilibrium, K represents the rate constant of the water intake through the hydrogel matrix, and n is the diffusion exponent for the swelling process. For swelling degrees up to 60% the coefficient n can be determined from the slope of the plot ln (SWt/SWe) = f[ln(t)] of the linearized form (3) of Equation (2), as follows:

(3) $$\ln\left(\frac{{\mathrm{SW}}_{t}}{{\mathrm{SW}}_{e}}\right)=\ln\left(K\right)+n\;\ln\left(t\right)$$

Knowing the value of the coefficient n, it can be established whether the swelling process was a first-order diffusion (n ≤ 5) or if it was a second- (n = 1.0) or third-order (0.5 < n < 1.0) non-Fickian diffusion. The plots’ linearity of Equation (4) is another way to determine if the swelling process follows the Fickian diffusion, as follows:

(4) $${\mathrm{SW}}_{t}=K\cdot t^{n}$$

For the second-order diffusion, Equation (5) is applied where integration between the limits SW *=* 0 to SW *=* SW_e_ and t *=* 0 to t results in the following Schott’s Equation (6):

(5) $$\frac{\mathrm{dSW}}{\mathrm{dt}}=K{\left(\mathrm{SW}-{\mathrm{SW}}_{e}\right)}^{2}$$

where: dSW/dt is the swelling rate, K is the rate constant (1/h), and SW and SW_e_ are the swelling degrees at time t and equilibrium, respectively.

(6) $$\frac{t}{\mathrm{SW}}=\frac{1}{{\mathrm{KSW}}_{e}^{2}}+\frac{1}{{\mathrm{SW}}_{e}}$$

The theoretical value for the SW_e_ and the rate constant K can be calculated from the slope of the Schott’s Equation t/SW = f(t), as follows:

(7) $${\mathrm{SW}}_{e}=\frac{1}{\mathrm{slope}}$$

and

(8) $$K=\frac{1}{\mathrm{Intercept}\;\times {\mathrm{SW}}_{e}^{2}}$$

### 4.4. H NMR Relaxometry

The molecular motion of water molecules throughout the polymer matrix was evaluated by measuring spin–spin ^1^H transverse relaxation times (T_2_) with a Bruker Minispec mq20 NMR spectrometer (Bruker, Karlsruhe, Germany). The T_2_ distributions were obtained by the inverse Laplace transform decays of the CPMG (Carr–Purcell–Meiboom–Gill), recording a total of 1000 echoes with echo times of 70 µs and 0.5 s recycle delay.

### 4.5. Antimicrobial Activity

The hydrogel samples were assessed against *Escherichia coli* ATCC 25922, *Salmonella enteritidis* ATCC 13076, and *Listeria monocytogenes* ATCC 19114 using the Kirby–Bauer diffusion method [42]. The purity of the inoculum was confirmed by plating on appropriate selective media and microscopic examination of the Gram-stained smear (Optika microscope, B252, M.A.D; Apparecchiature Scientifiche, Milan, Italy) [43,44]. Each bacterial strain was grown for 24 h on Mueller–Hinton agar medium (Oxoid Ltd., Basingstoke, Hampshire, England) at 37 °C. Several colonies were transferred in sterile saline solution (8.5 g/L) and adjusted to match the turbidity of McFarland 0.5 standard (1.5 × 10^8^ CFU/mL). One hundred microliters of inoculum (1.5 × 10^5^ CFU/mL) were dispersed over the entire surface of the Mueller–Hinton agar plate (Oxoid Ltd., Basingstoke, Hampshire, England) using a Drigalski spatula. The samples were cut into 5 mm diameter discs and, after the incubation period (24 h) at a temperature of 30 °C (hydrogels samples melt above this temperature), the zones of inhibition (mm) in the tested microbial strains were determined. A digital caliper was used to measure the inhibition zone diameter (mm). Two replicates were run for each hydrogel sample.

## Figures and Tables

**Table 1:** The degree of swelling at different times for hydrogel samples with increasing GO concentration (I, II, III, and IV), and control sample M.

Time (min)	Swelling Degree (g/g)				
	M	I	II	III	IV
60	1.6011	1.9960	1.8685	1.9846	1.8525
120	1.8555	2.1486	2.1509	2.3936	2.0603
240	2.2254	2.3820	2.5495	2.5556	2.3852
300	2.3353	2.4935	2.6507	2.6737	2.4908
1440	3.0603	2.9316	2.8867	2.9131	2.8918
2880	3.1581	3.1775	3.0415	2.9251	2.8670

**Table 2:** Calculated and experimental values for swelling degree (SW_e_) and for rate constant (K) based on Schott’s equation.

	Slope	SWe Calculated	SWe Experimental	Intercept	K
M	0.31	3.21	3.16	12.26	0.03
I	0.32	3.17	3.18	8.21	0.04
II	0.33	3.05	3.04	9.43	0.03
III	0.34	2.96	2.93	8.44	0.04
IV	0.34	2.91	2.87	8.56	0.04

**Table 3:** Diameters of inhibition zones (mm) for tested hydrogel samples.

Bacterial Strain	Sample Code *				
	M	I	II	III	IV
*Escherichia coli* ATCC 25922	0	0	9.7 ± 0.141	12.5 ± 0.212	13.7 ± 0.07
*Salmonella enteritidis* ATCC 13076	0	0	10.5 ± 0.035	12.5 ± 0.212	13.5 ± 0.141
*Listeria monocytogenes* ATCC 19114	0	0	0	10.5 ± 0.141	12.00 ± 0.00

* Values are expressed as mean of two replicates ± SD.

## Data Availability

Not applicable.
